# Does Hypoxia and Stress Erythropoiesis Compromise Cardiac Function in Healthy Adults? A Randomized Trial

**DOI:** 10.1186/s40798-022-00531-x

**Published:** 2022-11-05

**Authors:** Antonio L. Arrebola-Moreno, Rafael A. Casuso, Jacob Bejder, Thomas Christian Bonne, Andreas Breenfeldt Andersen, Jerónimo Aragón-Vela, Nikolai B. Nordsborg, Jesús R. Huertas

**Affiliations:** 1 Cardiology Department, HLA Inmaculada Hospital, Granada, Spain; 2 Department of Physiology, Institute of Nutrition and Food Technology, University of Granada, Granada, Spain; 3 Department of Nutrition, Exercise and Sports (NEXS), University of Copenhagen, Copenhagen, Denmark; 4 Department of Health Sciences, Universidad Loyola Andalucía, Sevilla, Spain

**Keywords:** Altitude, Haematology, Heart, Doping

## Abstract

**Objectives:**

To investigate whether recombinant human erythropoietin (rHuEPO) injections during an altitude training camp impact heart function.

**Methods:**

Thirty (12 women) moderately trained subjects stayed at 2320 m altitude for 4 weeks while training. Subjects were randomized to placebo (isotonic saline) or rHuEPO (20 IU/kg body weight) i.v. injections. Transthoracic echocardiography imaging was acquired 3 days after arrival to altitude and prior to the first placebo or rHuEPO injection as well as one day after the last rHuEPO injection three weeks later.

**Results:**

rHuEPO did not alter cardiovascular morphology parameters, systolic or diastolic function. In the placebo group, altitude exposure improved left ventricle (LV) systolic function due to an increased twist angle but rHuEPO had no additional effects. Pulmonary arterial systolic pressure was unaffected in either group. Notably, rHuEPO hampered LV untwist rate without affecting LV early filling.

**Conclusion:**

rHuEPO provided during mild altitude exposure does not cause any major effects on heart function. The observed alteration in LV untwist induced by rHuEPO is unlikely to have a meaningful clinical effect.

*Trial Registration* Registered on www.clinicaltrials.gov (NCT04227665).

**Supplementary Information:**

The online version contains supplementary material available at 10.1186/s40798-022-00531-x.

## Key Points

- Recombinant human erythropoietin (rHuEPO) administration during an altitude training camp does not clinically compromise cardiac function.
- While rHuEPO may alter left ventricle mechanics, this is likely due to a secondary response of its haematological effects.

## Introduction

Human sea-level residents frequently travel to altitude for recreational and work purposes [1]. In addition, athletes have for decades engaged in altitude training aimed at legally enhancing sea level performance via an increase in haemoglobin concentration and total haemoglobin mass [2–4]. However, reports exist to suggest that some athletes exploit altitude training camps to conceal a misuse of recombinant human erythropoietin (rHuEPO) [5]. The marked increase in endogenous erythropoietin (EPO) production during the first days even at moderate altitude [6, 7] in combination with prolonged (rHuEPO) treatment will increase activation of EPO receptors (EPOR).

EPOR are also expressed outside the hematopoietic system, including in cardiomyocytes [8, 9]. In rodents, a consequence of myocyte EPOR stimulation is improved haemodynamic characteristics [10, 11], reduced oxidative stress in a hypoxic situation [12] and remodelling [13]. Specifically, EPO administration improves LV function both during ischaemia‐reperfusion [14] and after myocardial infarction [15] in rats. In fact, it has been proposed that low dose rHuEPO administration would improve human cardiac disease through EPOR stimulation [16].

Human heart adapts its function and mechanics to preserve systolic function during altitude exposure. For instance, after several days to altitude exposure, there is a reduced stroke volume [17–19] which is not due to impaired myocardial function but likely due to a decreased ventricular filling pressure [20, 21]. Moreover, to compensate for a decreased diastolic filling, there is an increase in LV twist and untwisting rate thereby maintaining LV ejection fraction [17]. Importantly, a decreased LV diastolic filling is inversely related to pulmonary arterial systolic pressure [22]. However, it is currently unknown if the mild hypoxic stress in combination with rHuEPO treatment causes clinical relevant myocardial adaptations in healthy humans. The question is of relevance from a basic physiological understanding perspective, a perspective of protecting athletes’ health as well as a clinical perspective of inducing cardiac remodelling in patients. Therefore, here we aimed to study whether rHuEPO injections alter normal heart function and mechanics during mild altitude exposure.

## Methods

### Subjects

Thirty (12 females) moderately trained, sea level inhabitants and non-smoking subjects were recruited. None of the participants had been exposed to an altitude above 1,000 m for > 1 month or donated blood for > 3 months before the study. All the subjects gave their written informed consent to participate in a randomized, single-blinded, placebo-controlled study approved by the ethics committee of Copenhagen, Denmark (H-17036662), registered on www.clinicaltrials.gov (NCT04227665) and performed in accordance with the Declaration of Helsinki.

### Design

The present study was performed during the altitude trial arm of a larger study which aimed to determine biological markers for detection of blood doping at altitude [6]. Pre-data were acquired after three days upon arrival to moderate altitude (2320 m), before the first rHuEPO injection. Participants were randomly assigned either to blinded treatment with rHuEPO (*n* = 12, 5 females), consisting of intravenous injections of 20 IU × kg bw^−1^ epoetin alpha (Eprex, Janssen, Birkerød, Denmark), or to placebo (*n* = 18, 7 females) consisting of intravenous injections of ~ 0.3 mL isotonic saline (B. Braun, Melsungen, Germany). Participants wore a blindfold during injections to maintain blinding. Treatment started on the third day following arrival to altitude and was given every second day for three weeks, resulting in a total of eleven injections. Post-data were collected a day after the last injection. In this period, subjects trained daily including both resistance and endurance training. Details of the training program are reported elsewhere [23].

### Echocardiography

Transthoracic echocardiography was performed using a commercially available system (Epiq-7 Philips, Andover, Netherlands) with a X5-1, 1 to 6-mHz phased-array, transducer. Images were obtained from parasternal short-axis and apical four-chamber views with 2D frame rates of 60 to 100 frames/s and tissue Doppler frame rates > 100 frames/s. Off-line data analysis (Qlab, Version 12.0, Philips) was performed by a cardiologist blinded to group allocation.

### Two-Dimensional Echocardiography and Doppler Tissue Imaging

Two-dimensional (2D), pulsed-Doppler, and colour tissue Doppler imaging were performed from parasternal short-axis. Images were obtained at the same frame rate to facilitate subsequent LV torsion and rotation analysis. The LV ejection fraction was calculated offline from 3D images (3DQ System, Philips). We defined LV length as the end-diastolic length from the mitral valve hinge point plane to the most distal endocardium at the LV apex and it was measured in the apical 4-chamber view. Pulsed-wave Doppler images were used to measure aortic valve opening/closure and mitral valve opening. Isovolumetric relaxation time (IVRT) was calculated as the difference between mitral valve opening and aortic valve closure. The left and right ventricular outflow tract diameter was measured from the parasternal long-axis view and was used to calculate stroke volume as previously [24, 25]. Cardiac output (Q) was calculated as the product of stroke volume and heart rate. Longitudinal tissue velocities were measured off-line from 2D colour-coded tissue Doppler images and are reported as the average of 3 consecutive cardiac cycles.

### Speckle-Tracking Echocardiographic Analysis

Myocardial deformation was measured as previously described [24]. Global LV peak systolic longitudinal strain (GLS) was calculated in an 18-segment model from the apical two-chamber, three-chamber, and four-chamber views. Global circumferential strain (GCS) measured from parasternal short-axis views at the base and apex. LV twist, systolic twist velocity and untwisting velocity were calculated by subtracting the apical frame-by-frame data from the basal data. The percentage of untwist during isovolumic relaxation time (IVRT; %UTIVRT) was calculated as follows [21]: %UTIVRT = [(twist at aortic valve closure − twist at the end of IVRT)/twist at aortic valve closure] × 100.

### Statistics

Data are reported as mean ± standard deviation. Normality was evaluated by a Kolmogorov–Smirnov tests. Data were analysed using a 2 (Groups) × 2 (Time) repeated measures ANOVA, within subject design. When a significant interaction was reported, the model was adjusted with gender as covariate. Significant interactions were further evaluated by Student’s *t* test for independent samples (rHuEPO vs. placebo). Statistical significance was set at *P* < 0.05 and we considered *P* < 0.08 as a statistical tendency. Analyses were performed using SPSS version 20. Additional file 1: Fig. S1 was created with R.

## Results

### General Cardiovascular Characteristics

Heart rate, diastolic blood pressure and mean blood pressure were reduced in both placebo and rHuEPO groups following 3 weeks of moderate altitude exposure, whereas systolic blood pressure remained unchanged (Table 1). No time x treatment effect was observed for either of these parameters.

**Table 1:** Resting heart rate and blood pressure responses to rHuEPO administration during a high altitude training camp

	Pre		Post		Altitude training effect	Time × treatment effect
	Placebo (*n*)	rHuEPO (*n*)	Placebo (*n*)	rHuEPO (*n*)		
Heart rate	61.9 ± 9.47 (18)	63.1 ± 8.68 (12)	59.3 ± 11.77 (18)	53.9 ± 10.0 (12)	0.033	0.224
Systolic BP	121.8 ± 8.95 (18)	115.2 ± 8.74 (11)	121.2 ± 9.75 (18)	114.7 ± 8.25 (11)	0.757	0.952
Diastolic BP	72.0 ± 5.65 (18)	66.5 ± 5.63 (11)	65.4 ± 6.24 (18)	62.1 ± 5.63 (11)	0.003	0.529
Mean BP	88.6 ± 6.18 (18)	82.5 ± 8.25 (11)	84.0 ± 5.50 (18)	79.4 ± 5.57 (11)	0.027	0.678

Results are means ± SD (number of subjects) and 2 (group) × 2 (time) ANOVA *p* values.

*BP* Blood pressure, *rHuEPO* recombinant human erythropoietin

### Morphological Parameters

Table 2 summarizes several representative cardiac morphological parameters. No effects were observed for any of the measured parameters including end-diastolic volume, ejection fraction, end-systolic volume, LV mass, RV volume and left atrial mass following moderate altitude exposure or rHuEPO injections.

**Table 2:** Cardiac morphology and functional parameters

	Pre		Post		Altitude training effect	Training × treatment effect
	Placebo (*n*)	rhEPO (*n*)	Placebo (*n*)	rhEPO (*n*)		
*Morphological parameters*						
EDV (ml)	123.5 ± 31.02 (19)	124.0 ± 24.69 (12)	125.7 ± 31.51 (19)	124.5 ± 27.81 (12)	0.858	0.914
EF (%)	63.9 ± 4.07 (19)	64.1 ± 7.04 (12)	62.2 ± 5.94 (19)	61.6 ± 6.22 (12)	0.163	0.796
ESV (ml)	44.5 ± 12.24 (19)	44.8 ± 13.36 (12)	47.0 ± 11.88 (19)	48.2 ± 14.45 (12)	0.383	0.894
LV mass Dias(g)	172.3 ± 47.70 (19)	170.2 ± 35.09 (12)	165.5 ± 48.29 (19)	157.6 ± 28.62 (12)	0.387	0.797
LV mass syst (g)	107.2 ± 36.76 (19)	106.4 ± 28.72 (12)	109.1 ± 37.07 (19)	102.9 ± 23.07 (12)	0.930	0.753
LA vol (ml)	53.6 ± 17.74 (14)	53.1 ± 15.7 (8)	51.5 ± 13.81 (14)	52.5 ± 10.99 (8)	0.771	0.871
RV diam basal	38.1 ± 7.53 (18)	39.6 ± 5.68 (11)	38.2 ± 6.18 (18)	39.9 ± 4.90 (11)	0.889	0.944
*Systolic Function*						
Left ventricle						
LV SV	98.2 ± 24.87 (19)	105.3 ± 22.43 (12)	101.5 ± 26.08 (19)	109.6 ± 25.54 (12)	0.561	0.935
LV Q	6.03 ± 1.57 (18)	6.59 ± 1.38 (12)	5.92 ± 1.25 (18)	5.84 ± 1.53 (12)	0.260	0.420
${\text{S}^{\prime}}_{{{\text{mean}}}}$ (cm s^−1^)	8.33 ± 1.15 (17)	9.17 ± 1.17 (8)	8.71 ± 1.39 (17)	8.42 ± 0.97 (8)	0.621	0.131
Right ventricle						
RV SV	107.2 ± 41.79 (8)	72.6 ± 26.09 (8)	88. 4 ± 23.19 (8)	82.2 ± 16.62 (8)	0.649	0.168
RV Q	6.09 ± 2.53 (8)	4.44 ± 1.45 (8)	4.98 ± 1.80 (8)	4.38 ± 1.38 (8)	0.380	0.427
${\text{S}^{\prime}}_{{{\text{RV}}}}$ (cm s^−1^)	13.0 ± 2.34 (17)	13.5 ± 1.72 (11)	12.6 ± 1.65 (17)	12.1 ± 0.96 (11)	0.077	0.302
PAAT (ms)	138.6 ± 28.08 (10)	133.3 ± 24.66 (9)	130.0 ± 12.79 (10)	125.7 ± 13.83 (9)	0.241	0.946
*Diastolic Function*						
Left ventricle						
Peak E (cm s^−1^)	79.8 ± 16.29 (14)	78.2 ± 14.4 (10)	79.8 ± 16.04 (14)	76.3 ± 12.27 (10)	0.832	0.836
Peak A (cm s^−1^)	45.1 ± 11.48 (14)	50.4 ± 10.91 (10)	40.4 ± 7.85 (14)	41.4 ± 5.03 (10)	0.016	0.440
E/A ratio	1.85 ± 0.56 (14)	1.59 ± 0.31 (10)	2.02 ± 0.48 (14)	1.85 ± 0.27 (10)	0.097	0.724
${\text{E}^{\prime}}_{{{\text{mean}}}}$ (cm s^−1^)	14.0 ± 2.06 (17)	12.5 ± 2.18 (8)	14.4 ± 2.35 (17)	13.9 ± 1.55 (8)	0.146	0.419
${\text{A}^{\prime}}_{{{\text{mean}}}}$ (cm s^−1^)	6.59 ± 1.50 (17)	6.91 ± 1.60 (8)	5.96 ± 1.28 (17)	5.85 ± 0.90 (8)	0.047	0.597
${\text{E}^{\prime}}_{{{\text{mean}}}}$/${\text{A}^{\prime}}_{{{\text{mean}}}}$ ratio	2.24 ± 0.63 (17)	1.91 ± 0.61 (8)	2.52 ± 0.65 (17)	2.44 ± 0.49 (8)	0.033	0.500
${\text{E}}/{\text{E}^{\prime}}_{{{\text{lat}}}}$	5.14 ± 1.49 (13)	5.7 ± 1.39 (6)	5.1 ± 1.61 (13)	4.66 ± 0.60 (6)	0.343	0.317
Right ventricle						
${\text{E}^{\prime}}_{{{\text{RV}}}}$ (cm s^−1^)	12.8 ± 2.29 (16)	13.6 ± 2.64 (12)	11.7 ± 2.65 (16)	12.2 ± 2.31 (12)	0.066	0.873
${\text{A}^{\prime}}_{{{\text{RV}}}}$ (cm s^−1^)	8.4 ± 2.54 (16)	9.1 ± 2.41 (12)	7.7 ± 1.9 (16)	8.5 ± 2.5 (16)	0.319	0.950

Results are means ± SD (number of subjects) and 2 (group) × 2 (time) ANOVA *p* values. Abbreviations: EDV, end-diastolic volume; EF, ejection fraction; ESV, end-systolic volume; LV mass Dias, left ventricle mass in diastole; LV mass syst; left ventricle mass in systole; LA vol; left atrial volume; RV diam basal, right ventricle basal diameter; LV SV, left ventricle systolic volume; LV Q; left ventricle cardiac output; ${\text{S}^{\prime}}_{{{\text{mean}}}}$, mean S′ wave in the medial and lateral mitral annulus; RV SV, right ventricle systolic volume; RV Q, right ventricle cardiac output; ${\text{S}^{\prime}}_{{{\text{RV}}}}$, S′ wave in the lateral tricuspid annulus; PAAT, pulmonary arterial acceleration time; Peak E, peak E wave in the mitral flow; Peak A, peak A wave in the mitral flow; E/A ratio, peak E and peak A ratio; ${\text{E}^{\prime}}_{{{\text{mean}}}}$; mean lateral and medial E′ wave in the mitral annulus; ${\text{A}^{\prime}}_{{{\text{mean}}}}$, mean lateral and medial A′ wave in the mitral annulus; ${\text{E}}/{\text{E}^{\prime}}_{{{\text{lat}}}}$, ratio between E wave in the mitral flow and E′ wave in the lateral mitral annulus; ${\text{E}^{\prime}}_{{{\text{RV}}}}$, peak E′ wave in the lateral tricuspid annulus; ${\text{A}^{\prime}}_{{{\text{RV}}}}$, peak A′ wave in the lateral tricuspid annulus

### Left and Right Ventricle Systolic Function

Both LV stroke volume, Q and S′ mean wave in mitral annulus (${\text{S}^{\prime}}_{{{\text{mean}}}}$) remained unchanged throughout the analysed time points and were not influenced by rHuEPO (Table 2). Similarly, RV stroke volume and Q were unaffected by moderate altitude exposure and by rHuEPO. In addition, pulmonary artery acceleration time (PAAT) was unaffected by either condition. However, we observed a trend (*p* = 0.077) towards an increase in the S′ wave in the lateral tricuspid annulus (${\text{S}^{\prime}}_{{{\text{RV}}}}$) in the placebo group without being affected by rHuEPO (Table 2).

### Left and Right Ventricle Diastolic Function

We found that late filling (peak A wave) was reduced by moderate altitude exposure while left ventricle early filling was unchanged (peak E wave) (Table 2). In this regard, we have not observed a reduced early-to-late transmitral filling velocity (E/A ratio). Nevertheless, there was a reduced transmitral tissue velocity as we noted a reduced ratio between mean lateral and medial E′ wave in the mitral annulus (${\text{E}^{\prime}}_{{{\text{mean}}}}$) and ${\text{A}^{\prime}}_{{{\text{mean}}}}$ (mean lateral and medial A′ wave in the mitral annulus), mainly due to a decrease in ${\text{A}^{\prime}}_{{{\text{mean}}}}$ (Table 2). Finally, there was no effect on a filling pressure index (${\text{E}}/{\text{E}^{\prime}}_{{{\text{lat}}}}$) in the analysed subjects. Importantly, none of these parameters was altered by rHuEPO injections (Table 2). Regarding the RV, no rHuEPO effect was observed in transtricuspid tissue velocity, but moderate altitude exposure tended (*p* = 0.066) to decrease ${\text{E}^{\prime}}_{{{\text{RV}}}}$ (Table 2).

### Left Ventricle Mechanics

GCS and peak twist were increased during altitude exposure without an effect of rHuEPO (Table 3). No effect of altitude was observed for LV relaxation parameters such as % Untwist under IVRT or peak untwisting velocity. However, an interaction effect was observed for peak untwisting velocity (Table 3). We found a similar interaction (*p* = 0.039) when the model was adjusted for the gender of the subjects (Additional file 1). Post hoc test revealed a lower peak untwisting velocity in rHuEPO subjects if compared with placebo subjects at post (Additional file 1).

**Table 3:** Left ventricle ventricular strain and twisting mechanics

	Pre		Post		Altitude training effect	Time × treatment effect
	Placebo (*n*)	rHuEPO (*n*)	Placebo (*n*)	rHEPO (*n*)		
GCS (%)	24.3 ± 2.91 (19)	25.1 ± 3.43 (12)	27.9 ± 4.16 (19)	26.8 ± 2.80 (12)	0.005	0.280
GLS (%)	18.9 ± 2.15 (19)	18.3 ± 2.13 (12)	18.8 ± 1.94 (19)	19.0 ± 1.99 (12)	0.545	0.441
Twist peak (º)	5.03 ± 2.14 (18)	4.78 ± 3.14 (11)	6.32 ± 2.31 (18)	6.67 ± 1.91 (11)	0.020	0.934
Peak twisting velocity (º/seg)	50.4 ± 23.84 (18)	51.2 ± 24.75 (11)	51.4 ± 21.67 (18)	46.0 ± 11.38 (11)	0.725	0.599
% Untwist under IVRT	55.0 ± 17.54 (17)	62.4 ± 22.89 (12)	65.4 ± 19.66 (17)	54.9 ± 22.89 (12)	0.792	0.107
Peak untwisting velocity (º/seg)	81.5 ± 32.35 (18)	97.7 ± 51.00 (12)	91.8 ± 33.82 (18)	67.3 ± 26.57 (12)*	0.296	0.038
Untwist velocity/peak twist	22.5 ± 20.57 (17)	24.7 ± 17.03 (12)	16.7 ± 13.83 (17)	12.6 ± 8.54 (12)	0.040	0.464

Results are means ± SD (number of subjects) and 2 (group) × 2 (time) ANOVA *p* values.

*GCS* global circumferential strain, *GLS* global longitudinal strain, *IVRT* interventricular relaxation time.

*p < 0.05 vs Placebo Post

## Discussion

The present study analysed heart function and mechanics during moderate altitude exposure in subjects administered with rHuEPO or isotonic saline. Here, we show that rHuEPO injections during an altitude training camp do not have a clinically relevant impact on heart function. We observed an altered untwisting velocity induced by rHuEPO which was concomitant with unchanged early diastolic filling and filling pressures, thus suggesting that rHuEPO does not alter myocardial mechanics. Instead, this effect may reflect that subjects receiving rHuEPO do not need to activate a compensatory mechanism such as untwisting in order to maintain a given Q.

### Methodological Considerations

Several important methodological considerations should be taken into account when interpreting our data. Indeed, preliminary data were collected during acclimatisation (i.e., 3 days after arrival to 2230 m), immediately before rHuEPO and placebo injections. This is of importance as most of the acute effects of hypoxia on the heart are evident within the first 4 days following exposure [17]. For instance, early (E wave) and late (A wave) left ventricular filling are not altered after 2 days of HA exposure [26] while a reduced early ventricular filling is evident after 2–6 days in hypoxia [17]. In the present study, we observed an increment of the E/A ratio by means of reduced late filling. While this seems to be inconsistent with the literature [20, 21, 25, 27], this is probably a result of a long-term normalization following altitude acclimatization. In agreement, a rapid increase in late filling has been reported upon arrival to 4320 m above sea level [17]. In this regard, it must be highlighted that the present study design does not allow to reach conclusions on the effects of mild hypoxic exposure on heart function but may provide indications of longer term adaptation to mild hypoxia.

In addition, PAAT, an accurate marker of pulmonary arterial systolic pressure [28], was unaltered under our experimental conditions. Thus, although inferred from an indirect measurement, pulmonary arterial systolic pressure appears unaltered by moderate altitude as well as by rHuEPO.

### Left Ventricle Filling and Mechanics in Response to rHuEPO

The store and release of energy through elastic components are a key process linking systole and diastole. The diastole phase consists of two phases: initially, due to the stored energy of the elastic component, the pressure gradient between the atria and the ventricles is high and ventricular filling is rapid (the phase of rapid early filling). Under normal circumstances, about 70% of ventricular filling occurs during this phase. As diastole progresses, ventricular pressure rises and the rate of filling slows (i.e. diastasis phase). The final ~ 25% of filling during ventricular diastole results from atrial contraction (i.e. atrial systole phase) [29].

Untwisting occurs during the diastole phase of rapid early filling and is directly influenced by the relaxation of cardiomyocytes and the release of potential energy stored during systole [30]. Interestingly, we found that untwisting velocity decreased in the rHuEPO group following 3 weeks of altitude exposure. Moreover, while placebo group increased %UTIVRT by ~ 20%, the rHuEPO group showed a ~ 13% decrement. There are two potential reasons for these observations. In one hand, rHuEPO may alter early diastolic filling during HA exposure; however, ${\text{E}^{\prime}}_{{{\text{mean}}}}$ was not altered by rHuEPO thus excluding a potential alteration in myocardial relaxation. Moreover, we found no effect of rHuEPO in early diastolic filling (E wave) or filling pressures. Therefore, it seems unlikely that rHuEPO alterations of myocardial mechanics result in a modulation of early LV filling. However, further studies perhaps examining subendocardial and subpericardial are needed in order to elucidate whether rHuEPO could differently affect either layer during altitude exposure.

On the other hand, rHuEPO is better known for its effect outside the cardiovascular system in humans. For instance, increased red cell production, angiogenesis, myogenesis, shift in muscle fibre types, and oxidative enzyme activities in skeletal muscle have been reported [31] that may also lead to improve maximal oxygen uptake [32]. In this scenario, rHuEPO may help to maintain a given Q through haematological and metabolic mechanisms without an increased untwisting rate. In agreement with this hypothesis, subjects in the rHuEPO group showed higher erythropoietic response than subjects in placebo group [6]. Therefore, while the rHuEPO dose used in the present study is of physiological relevance [32], it seems unlikely to alter cardiomyocyte function.

Finally, it has been reported that untwist velocity increases immediately in response to altitude exposure, an effect that is maintained for 4 days, but which is normalized to sea levels after 6 days [17]. Thus, the lack of effect of altitude on this heart mechanics parameter could be easily explained by the fact that our preliminary measurements were obtained 3 days after arrival to altitude. In contrast, we were able to detect the common observation of increased twist angle in response to altitude exposure [17, 21, 25, 29, 33]. In this regard, it is important to highlight that endurance training decreases twist angle without altering untwist velocity [34]. Thus, as our sample was already trained subjects then, an increased twist angle would be easier to detect even with the design limitations discussed above.

## Conclusions

Here, we show that low dose rHuEPO injections provided during mild hypoxic exposure do not cause any major effects on heart function. Thus, no immediate health hazards are obvious for athletes training at altitude and no obvious beneficial effects of mild hypoxic exposure and/or rHuEPO treatment can be expected as evaluated on a young symptom free healthy population.

## Supplementary Information

**Additional file 1. Supplementary Figure 1**. Untwist velocity in response to mild altitude and rHuEPO injections. p values denotes post hoc test (Student´s t) analysis following a significant 2 (Groups) x 2 (Time) repeated measures ANOVA, within subject design corrected and corrected for Gender (p = 0.039 see main text).

## Data Availability

All data related to this study are presented within the manuscript.

## Abbreviations

${\text{A}^{\prime}}_{{{\text{mean}}}}$: Mean lateral and medial A′ wave in the mitral annulus
${\text{A}^{\prime}}_{{{\text{RV}}}}$: Peak A′ wave in the lateral tricuspid annulus
BP: Blood pressure
EDV: End-diastolic volume
EF: Ejection fraction
ESV: End-systolic volume
LV mass Dias: Left ventricle mass in diastole
E/A ratio: Peak E and peak A ratio
${\text{E}^{\prime}}_{{{\text{mean}}}}$: Mean lateral and medial E′ wave in the mitral annulus
${\text{E}}/{\text{E}^{\prime}}_{{{\text{lat}}}}$: Ratio between E wave in the mitral flow and E′ wave in the lateral mitral annulus
${\text{E}^{\prime}}_{{{\text{RV}}}}$: Peak E′ wave in the lateral tricuspid annulus
GCS: Global circumferential strain
GLS: Global longitudinal strain
IVRT: Interventricular relaxation time
LV mass syst: Left ventricle mass in systole
LA vol: Left atrial volume
RV diam basal: Right ventricle basal diameter
LV SV: Left ventricle systolic volume
LV Q: Left ventricle cardiac output
${\text{S}^{\prime}}_{{{\text{mean}}}}$: Mean S′ wave in the medial and lateral mitral annulus
RV SV: Right ventricle systolic volume
RV Q: Right ventricle cardiac output
${\text{S}^{\prime}}_{{{\text{RV}}}}$: S′ wave in the lateral tricuspid annulus
PAAT: Pulmonary arterial acceleration time
Peak E: Peak E wave in the mitral flow
Peak A: Peak A wave in the mitral flow
Q: Cardiac output
rHuEPO: Recombinant human erythropoietin
