# Supplementary material for: Does Hypoxia and Stress Erythropoiesis Compromise Cardiac Function in Healthy Adults? A Randomized Trial
Source: Sports Med Open. 2022 Nov 5;8:137. doi: 10.1186/s40798-022-00531-x (PMC9637068; doi:10.1186/s40798-022-00531-x)
Supplement: Supplementary file 1 — Additional file 1. Supplementary Figure 1. Untwist velocity in response to mild altitude and rHuEPO injections. p values denotes post hoc test (Student´s t) analysis following a significant 2 (Groups) x 2 (Time) repeated measures ANOVA, within subject design corrected and corrected for Gender (p = 0.039 see main text). [file 40798_2022_531_MOESM1_ESM.docx]

**
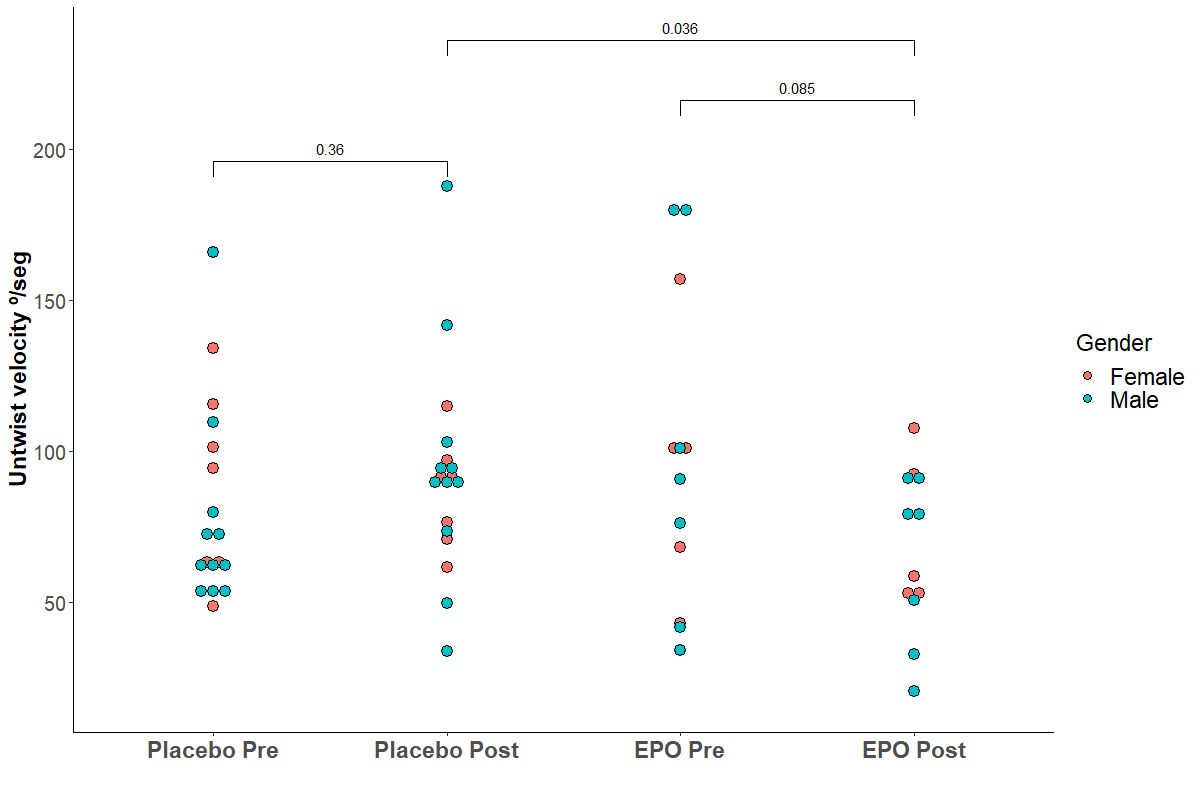
**

**Supplementary Figure 1.** Untwist velocity in response to mild altitude and rHuEPO injections. p values denotes post hoc test (Student´s *t*) analysis following a significant 2 (Groups) x 2 (Time) repeated measures ANOVA, within subject design corrected and corrected for Gender (*p* = 0.039 see main text).
